# Ultranarrow polaritonic cavities formed by one-dimensional junctions of two-dimensional in-plane heterostructures

**DOI:** 10.1515/nanoph-2025-0467

**Published:** 2025-11-27

**Authors:** Seojoo Lee, Ji-Hun Kang

**Affiliations:** School of Applied and Engineering Physics, Cornell University, Ithaca, NY 13853, USA; The Institute of Basic Science, Korea University, Seoul 02841, Republic of Korea; Department of Optical Engineering, Kongju National University, Cheonan 31080, Republic of Korea; Department of Future Convergence Engineering, Kongju National University, Cheonan 31080, Republic of Korea; Institute of Application and Fusion for Light, Kongju National University, Cheonan 31080, Republic of Korea

**Keywords:** surface polaritons, metasurfaces, polaritonic resonators, two-dimensional materials, spoof surface polaritons

## Abstract

We propose two-dimensional (2D) in-plane heterostructures, composed of a 2D crystal adjoining a perfect electric conductor (PEC) plane, that enable ultranarrow polaritonic resonant cavities. Specifically, we theoretically investigate the interaction of 2D surface polaritons (2DSPs) with the junction between the 2D crystal and a PEC plane. We reveal that when 2DSPs are strongly confined, the reflected 2DSPs experience a phase shift of 3*π*/4, which exhibits *π*/2 deviation from the so-called edge reflection value. This non-trivial phase shift is shown to play a crucial role in enabling resonant cavities whose size can be far smaller than the wavelength of the 2DSPs. Furthermore, we demonstrate that the spatial dimensionality of our heterostructure allows a direct mapping to metasurface-based heterostructures, where the 2D crystal is replaced by a metasurface supporting spoof surface polaritons (SSPs). This correspondence extends the feasibility of our concept to SSP-based resonators and broadens the accessible frequency range into the terahertz and microwave regimes. Our work provides not only deeper insight into low-dimensional polariton optics but also a design strategy for ultracompact polaritonic metaresonators.

## 1 Introduction

Two-dimensional surface polaritons (2DSPs) are hybrid quasiparticles formed by photons coupled to collective excitations (plasmons, phonons, etc.) in atomically thin crystals [1], [2], [3], [4], [5], [6]. By capturing electromagnetic waves at deep sub-wavelength scales, they yield intense local fields and enable novel nanophotonic functionalities, from enhanced light–matter interactions to electrically tunable ultracompact modulators [7], [8], [9], [10], [11], [12], [13], [14]. Controlling 2DSPs is therefore essential for polariton-based applications, and a fundamental strategy to achieve such control is to utilize their interaction with spatially varying boundaries of the supporting 2D crystal [15], [16], [17], [18], [19], [20]. The simplest example is the interaction between 2DSPs and the crystal’s edge. In this case, it is well established that when the momentum of the 2DSPs strongly exceeds that of free-space photons, they experience nearly total internal reflection with an accompanying phase shift of *π*/4 [21], [22], [23], [24]. More complex geometries have also been considered: for instance, a finite gap within the 2D crystal enables reflection and tunneling of 2DSPs [16], [17], [25], while placing plasmonic nanoplates on the crystal surface leads to reflection and phase modulation induced by their interaction [15], [26]. Recently, rigorous analytic theories have further advanced this line of study, revealing principles for manipulating 2DSPs on 2D materials that correspond to the classical laws governing light manipulation in three-dimensional free space. Significant progress has been made, including the formulation of Fresnel coefficients for 2DSPs [22], [23]. These studies collectively establish that engineering the boundaries of 2D crystals is at the core of manipulating 2DSPs, implying that diverse boundary-engineering strategies could enable broad control of light within the reduced dimensionality. At the same time, because the polaritonic response of widely studied 2D crystals, such as graphene and hexagonal boron nitride, occurs mostly in the infrared, it is important to explore ways to extend this capability to other spectral regions.

In this work, we propose an in-plane 2D polaritonic heterostructure of a 2D crystal and a 2D perfect electric conductor (PEC), whose interface is a one-dimensional (1D) junction. Specifically, we investigate how 2DSPs interact with this junction, and reveal an anomalous reflection phase response that enables ultranarrow polaritonic cavities. Using the coupled-mode theory, we obtain a closed-form reflection coefficient for strongly confined polaritons. In the limit of strong confinement, 2DSPs reflect with nearly unity amplitude and a phase shift of 3*π*/4, which is a *π*/2 additional shift beyond the typical edge-reflection phase. This phase shift allows two such junctions to form a resonant cavity even when spaced far below the polariton wavelength. Moreover, by using the in-plane nature of the system, we propose a practical analog of the ideal crystal/PEC system: a metasurface supporting spoof surface polaritons (SSPs) patterned between parallel PEC plates. This metasurface heterostructure is shown to preserve the resonant behavior of the original 2D system, while offering broad tunability across terahertz to microwave frequencies. Our results provide a new strategy for phase-engineering polaritonic interfaces and open the door to ultracompact polaritonic resonators for nanophotonic and plasmonic devices.

## 2 Analytic theory – orthonormal eigenfunctions for the problem space

**Figure 1:**
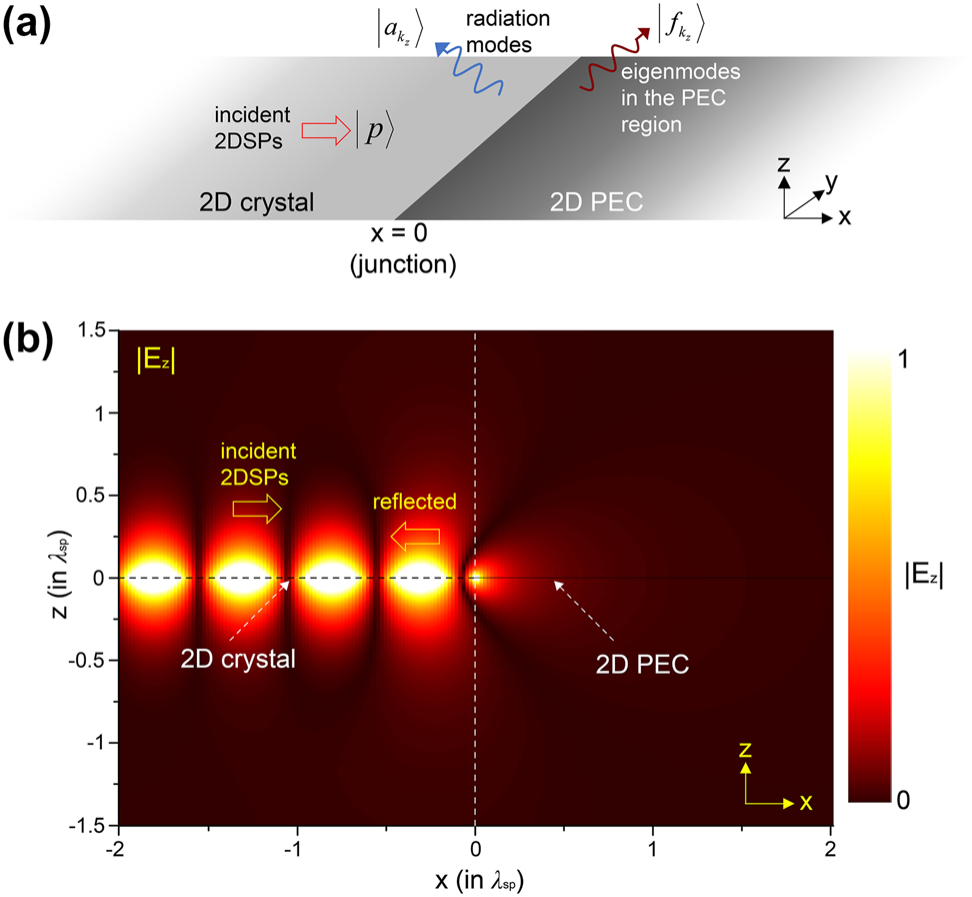
Interaction of 2DSPs with a one-dimensional junction between a 2D crystal and a 2D PEC plane. (a) Schematic of the system. 2DSPs, uniform in the *y*-direction, are incident normally to the junction. (b) Analytically calculated field map of |*E*_*z*_|, showing interference patterns resulting from the interaction between the incident and reflected 2DSPs. The momentum of 2DSPs is set to *p*_*x*_ = 50*k*_0_.

Consider a semi-infinitely wide, infinitesimally thin 2D crystal located at *z* = 0 and *x* ≤ 0. At *x* ≥ 0, there is the 2D PEC plane located at *z* = 0, as shown in Figure 1(a). Strongly-confined 2DSPs, excited far to the left of the 1D junction between the 2D crystal and the PEC plane, propagate along the +*x*-direction with momentum *p*_*x*_, which is larger than the photon momentum *k*_0_. We assume that the 2D crystal is lossless for clear definition of the orthonormal eigenfunctions of the system. The objective here is to analyze the interaction between the incident 2DSPs and the 1D junction at *x* = 0. The interaction involves diffraction of 2DSPs, resulting in the excitation of all possible orthogonal eigenmodes in the system. Let $\left|p\right\rangle$ the orthonormal eigenfunction of 2DSPs. It has been discussed that 2DSPs exhibit anti-symmetric field distribution in the *z*-direction. Specifically, the real-space representation of 2DSPs can be written as

(1) $$\left\langle z\left|\right.p\right\rangle =\sqrt{-ip_{z}}\frac{z}{\left|z\right|}{\text{e}}^{\text{i}p_{z}|z|},$$

with $p_{z}\equiv \sqrt{k_{0}^{2}-p_{x}^{2}}$ the momentum of 2DSPs in the *z*-direction. Because of the anti-symmetric nature of 2DSPs and symmetric configuration of the system in the *z*-direction, all the consequent results from the interaction are restricted to be anti-symmetric. That is, we can consider only the anti-symmetric eigenfunctions in the system. In the crystal region (*x* ≤ 0), the remaining orthonormal eigenfunction is surface-unbound anti-symmetric radiation mode $\left|a_{k_{z}}\right\rangle$ , whose the real-space representation is given by

(2) $$\left\langle z\left|\right.a_{k_{z}}\right\rangle \equiv \frac{1}{\sqrt{2\pi }}\sqrt{\frac{p_{z}^{2}}{p_{z}^{2}-k_{z}^{2}}}\left[\frac{z}{\left|z\right|}\frac{k_{z}}{p_{z}}\cos\left(k_{z}z\right)+i\sin\left(k_{z}z\right)\right].$$

One can readily show the orthonormality of the eigenfunctions: $\left\langle p\left|\right.p\right\rangle \equiv \int_{-\infty }^{\infty }dz{\left|\left\langle z\left|\right.p\right\rangle \right|}^{2}=1$ , $\left\langle a_{k_{z1}}\left|\right.a_{k_{z}}\right\rangle =\delta \left(k_{z}-k_{z1}\right)$ , and $\left\langle p\left|\right.a_{k_{z}}\right\rangle =0$ with *δ* the Dirac delta function. In the PEC plane region (*x* ≥ 0), the orthonormal eigenfunction $\left|f_{k_{z}}\right\rangle$ is given under the requirement of the PEC boundary condition at *z* = 0^±^, and the anti-symmetricity in the *z*-direction. Then, the real-space representation of the orthonormal eigenfunction can be found as

(3) $$\left\langle z\left|\right.f_{k_{z}}\right\rangle =\frac{1}{\sqrt{2\pi }}\frac{z}{\left|z\right|}\cos\left(k_{z}z\right).$$

It should be noted that the interaction between 2DSPs and the junction at *x* = 0 is solely determined by the dependencies between the eigenfunctions in two different regions. The reflection of 2DSPs is equivalent to the recoupling of $\left|p\right\rangle$ by $\left|f_{k_{z}}\right\rangle$ , where $\left|f_{k_{z}}\right\rangle$ can be initially induced by incident $\left|p\right\rangle$ via non-orthogonal dependency between the two eigenfunctions. Specifically, inner products between functions in the two regions can be found as

(4) $$\begin{array}{cc} \left\langle a_{k_{z1}}\left|\right.f_{k_{z}}\right\rangle & \equiv \overset{\infty }{\underset{-\infty }{\int }}dz\left\langle a_{k_{z1}}\left|\right.z\right\rangle \left\langle z\left|\right.f_{k_{z}}\right\rangle \\ & =W_{a,f}\left(k_{z1}\right)\delta \left(k_{z}-k_{z1}\right)+K_{a,f}\left(k_{z};k_{z1}\right),\\ \left\langle p\left|\right.f_{k_{z}}\right\rangle & \equiv \overset{\infty }{\underset{-\infty }{\int }}dz\left\langle p\left|\right.z\right\rangle \left\langle z\left|\right.f_{k_{z}}\right\rangle =W_{p,f}\left(k_{z}\right). \end{array}$$

Here, $W_{a,f}\left(k_{z}\right)\equiv -\frac{k_{z}}{p_{z}}\sqrt{\frac{p_{z}^{2}}{p_{z}^{2}-k_{z}^{2}}}$ defines the coupling between $\left|a_{k_{z1}}\right\rangle$ and $\left|f_{k_{z}}\right\rangle$ , while that between $\left|p\right\rangle$ and $\left|f_{k_{z}}\right\rangle$ is defined by $W_{p,f}\left(k_{z}\right)\equiv -\sqrt{-ip_{z}}\frac{1}{\sqrt{2\pi }}\frac{2ip_{z}}{k_{z}^{2}-p_{z}^{2}}$ . The kernel $K_{a,f}\left(k_{z};k_{z1}\right)\equiv \frac{1}{2\pi }\sqrt{\frac{p_{z}^{2}}{p_{z}^{2}-k_{z1}^{2}}}\frac{2ik_{z1}}{k_{z}^{2}-k_{z1}^{2}}$ describes a non-vanishing dependency between $\left|f_{k_{z}}\right\rangle$ and $\left|a_{k_{z1}}\right\rangle$ with *k*_*z*1_ ≠ *k*_*z*_.

Now, we are ready to describe dynamic procedure of the interaction. The *y*-component of the magnetic fields in the two regions can be written as

(5) $$\begin{array}{cc} \left|H_{y}^{x\leq 0}\left(x\right)\right\rangle & =\left({\text{e}}^{\text{i}p_{x}x}-Re^{-ip_{x}x}\right)\left|p\right\rangle \\ & \;-\overset{\infty }{\underset{-\infty }{\int }}dk_{z}\alpha_{k_{z}}\left|a_{k_{z}}\right\rangle e^{-ik_{x}x},\\ \left|H_{y}^{x\geq 0}\left(x\right)\right\rangle & =\overset{\infty }{\underset{-\infty }{\int }}dk_{z}\phi_{k_{z}}\left|f_{k_{z}}\right\rangle {\text{e}}^{\text{i}k_{x}x}. \end{array}$$

Here, $k_{x}\equiv \sqrt{k_{0}^{2}-k_{z}^{2}}$ , *R* is the reflection coefficient of $\left|p\right\rangle$ , and *α* and *ϕ* respectively are amplitudes of $\left|a_{k_{z}}\right\rangle$ and $\left|f_{k_{z}}\right\rangle$ . The corresponding *z*-components of the electric field can be obtained from the Maxwell equations. By applying the boundary conditions at *x* = 0, equivalent to the continuity of tangential components of electric and magnetic fields, we have

(6) $$\begin{array}{c} \left(1-R\right)\left|p\right\rangle -\overset{\infty }{\underset{-\infty }{\int }}dk_{z}\alpha_{k_{z}}\left|a_{k_{z}}\right\rangle =\overset{\infty }{\underset{-\infty }{\int }}dk_{z}\phi_{k_{z}}\left|f_{k_{z}}\right\rangle ,\\ p_{x}\left(1+R\right)\left|p\right\rangle +\overset{\infty }{\underset{-\infty }{\int }}dk_{z}k_{x}\alpha_{k_{z}}\left|a_{k_{z}}\right\rangle =\overset{\infty }{\underset{-\infty }{\int }}dk_{z}k_{x}\phi_{k_{z}}\left|f_{k_{z}}\right\rangle . \end{array}$$

Note that the left- and right-hand sides of each equation in Eq. (6) are expressed in different vector spaces. Explicit constraints arising from the continuity condition can be examined by projecting Eq. (6) onto each of the two spaces, thereby yielding coupled integral equations that can be treated using various methods [17], [22], [27], [28], [29]. Here, we adopt the Born approximation (BA). After some manipulations, the reflection coefficients obtained from the zero-th and first BAs, for instance, can be expressed as (see Supplementary Material)

(7) $$\begin{array}{c} R^{\left(0\right)}=\frac{I^{\left(0\right)}-1}{I^{\left(0\right)}+1},I^{\left(0\right)}\equiv \overset{\infty }{\underset{-\infty }{\int }}dk_{z}\frac{k_{x}}{p_{x}}{\left|W_{p,f}\left(k_{z}\right)\right|}^{2},\\ R^{\left(1\right)}=\frac{I^{\left(1\right)}-1}{I^{\left(1\right)}+1},I^{\left(1\right)}\equiv \overset{\infty }{\underset{-\infty }{\int }}dk_{z}\frac{k_{x}}{p_{x}}\frac{{\left|W_{p,f}\left(k_{z}\right)\right|}^{2}}{1+{\left|W_{a,f}\left(k_{z}\right)\right|}^{2}}. \end{array}$$

## 3 Reflection amplitudes and anomalous phase shifts

**Figure 2:**
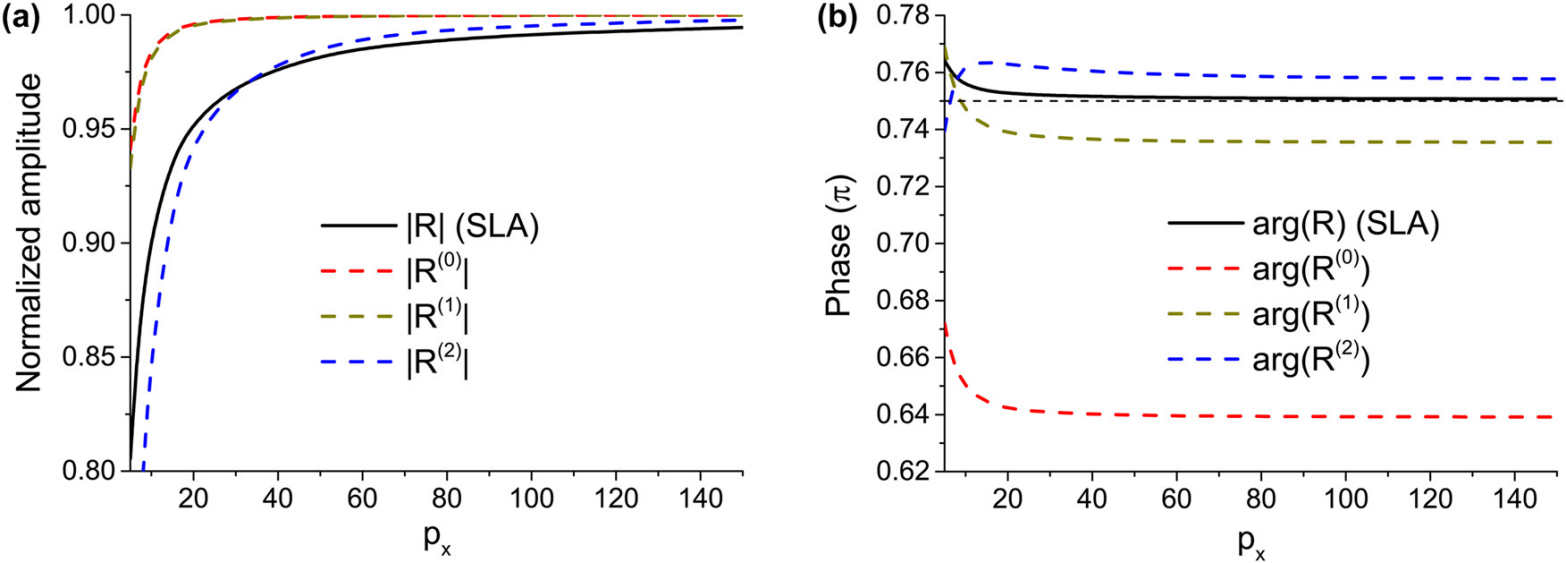
Analytically calculated 2DSP reflection coefficient. (a) Amplitude and (b) phase of *R* as a function of *p*_*x*_, obtained by using zero-th, first, and second BA. The black curves are obtained by using the SLA method. In (b), black horizontal dashed line denotes 0.75*π*. In the calculations, we set *k*_0_ = 1.

So far, we have obtained the reflection coefficient of 2DSPs using BA. The amplitude and phase of the reflected 2DSPs as a function of *p*_*x*_ are shown in Figure 2. For numerically accurate reference, we also computed *R* by adopting the super-lattice approximation (SLA) to solve the coupled integral equations in a semi-analytic manner by quantizing the integral parts [21]. In Figure 2(a), we can see that, as *p*_*x*_ increases, the reflection amplitudes from both SLA and BA approach 1, indicating that the reflection coefficient tends towards total internal reflection, similar to the edge-reflection case. For *p*_*x*_ >> *k*_0_, the 2DSPs are strongly bound within the 2D crystal. Upon encountering an abrupt transition to a non-propagating surface at the junction, they experience strong reflection due to large momentum mismatch between $\left|p\right\rangle$ and $\left|f_{k_{z}}\right\rangle$ , resulting in nearly total internal reflection.

Shown in Figure 2(b) is phase shift associated with the reflection. As *p*_*x*_ increases, the phase shift is shown to saturate to specific values depending on the approximation methods. In particular, SLA predicts that the phase approaches an anomalous value of 3*π*/4. Specific closed forms of the phase shift are available from the other approximations. For *p*_*x*_ >> *k*_0_, the zero-th coupling factor *I* ^(0)^ in Eq. (7) can be approximated as *I* ^(0)^ ≈ 2*i*/*π*. This gives rise to |*R* ^(0)^| ≈ 1 and arg(*R* ^(0)^) ≈ tan^−1^(4*π*/(4 − *π* ^2^)) + *π* ≈ 0.639*π*, exhibiting more than 15 % discrepancy compared to the result from SLA. However, for the first BA, *I* ^(1)^ can be approximated as *I* ^(1)^ ≈ 2*i*ln(2)/*π*. This yields |*R* ^(1)^| ≈ 1 and arg(*R* ^(1)^) ≈ 0.735*π*, which is deviated only about 2 % from 3*π*/4. One can find that *R* ^(2)^, reflection coefficient by the second BA, yields |*R* ^(2)^| ≈ 1 and arg(*R* ^(2)^) ≈ 0.757*π*, which gets even closer to 3*π*/4 (see Supplementary Material). For *p*_*x*_ < 10*k*_0_, however, we can see that the second BA does not show monotonic decrease of the phase shift with increasing *p*_*x*_, implicating a necessity of higher BA.

It is worth noting that 3*π*/4 is an intriguing value, as it exhibits an *exact π*/2 difference from the *π*/4 phase shift emerging in the edge reflection. The origin of such phase shifts is ascribed to induced evanescent waves near the junction [15]. Moreover, together with the nearly total internal reflection behavior, the phase shifts are interpreted in terms of Goos–Hänchen effect [21], [22]. This implies that the junction may behave as a reactive boundary capable of temporary storing electromagnetic energy. From this perspective, the phase response could be described more intuitively by introducing an effective impedance formalism for the polariton interface. This could give a deeper physical meaning of the *π*/2 difference, a question that should be done in future work.

## 4 Resonant polaritonic cavity

**Figure 3:**
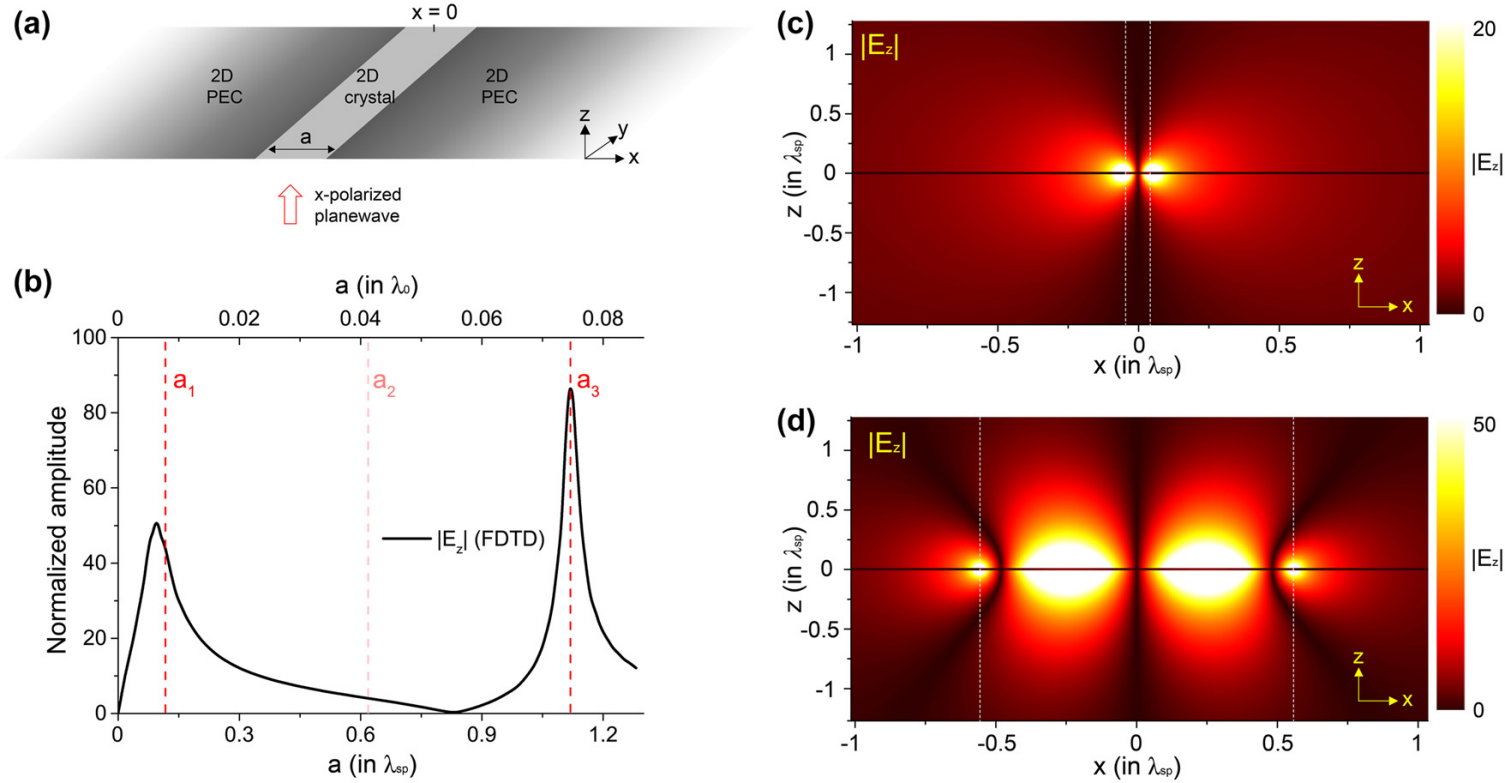
Resonant polaritonic cavity. (a) Schematic of the system with two junctions. The width of the 2D crystal is *a*, defining the separation between the junctions. An *x*-polarized planewave is incident upon the system normally from *z* < 0. (b) FDTD-calculated |*E*_*z*_| with varying *a*. The amplitude is taken at the surface of the left junction (*x* = −*a*/2). The amplitude is normalized by that of the incident. Three red vertical dashed lines denote the positions of *a*_1_, *a*_2_, and *a*_3_, obtained from Eq. (8). (c, d) FDTD-calculated *x*–*z* field maps of |*E*_*z*_| for (c) the left peak (*a* ≈ *a*_1_) and (d) the right peak (*a* ≈ *a*_3_) of (b). The vertical dotted lines indicate the positions of the two junctions. In FDTD calculations, we set *h* = *λ*_0_/1,500 with *λ*_sp_ = *λ*_0_/15, where *h* is the thickness of the 2D crystal and PEC planes.

In a practical point of view, the significance of 3*π*/4 can be found in the cavity resonance. For a cavity defined by two identical boundaries separated by distance *a*, its resonance condition can be written as *a*_*m*_ = *λ*(*m* − *θ*/*π*)/2, where *λ* is the wavelength of wave inside the cavity, *m* = 0, 1, 2…, and *θ* is the reflection phase shift of by the boundary. The phase *θ* plays a critical role in determining the size of a resonant cavity, or resonance wavelength for a fixed cavity. If we define a cavity for 2DSPs by two junctions between 2D crystal and PEC plane with the separation *a*, as shown in Figure 3(a), we can set *θ* ≡ arg(*R*) ≈ 3*π*/4 and the resonance condition can be predicted as

(8) $$\begin{array}{ll} a_{m} & =\frac{\lambda_{sp}}{2}\left(m-\frac{\arg\left(R\right)}{\pi }\right)\approx \frac{\lambda_{sp}}{2}\left(m-\frac{3}{4}\right),\\ & \;\;\left(m=1,2,3...\right). \end{array}$$

This suggests that, owing to the anomalous phase shift of 2DSPs at the junction, the fundamental resonance (*m* = 1) can emerge when the cavity size is given by *a*_0_ ≈ *λ*_sp_/8, which is much narrower than that of a cavity with two perfect-mirror-like boundaries (*a*_0_ ≈ *λ*/2). To check the validity of the resonant polaritonic cavity, we performed numerical calculations by using the finite-difference time-domain (FDTD) method. 2DSPs are excited by an *x*-polarized planewave incident normally upon the system from *z* < 0. Since implementing an exact 2D material with zero thickness is challenging in FDTD, we considered that both 2D crystal and PEC plane have a finite thickness *h* = *λ*_0_/1,500 with *λ*_sp_ = *λ*_0_/15. Shown in Figure 3(b) is the FDTD-calculated amplitude of *E*_*z*_, taken at the left junction (*x* = −*a*/2) with varying *a*. We can see that there are two resonances around *a* = *a*_1_ and *a*_3_, as predicted by Eq. (8). The right peak at *a* = *a*_3_ shows an excellent agreement between the FDTD result and Eq. (8), but for the left peak near *a* = *a*_1_, we can observe about 15 % discrepancy. We ascribe this discrepancy to the interaction between the junctions mediated by the radiation modes $\left|a_{k_{z}}\right\rangle$ . This cannot be captured by Eq. (8), but is expected to play a non-negligible role when *a* << *λ*_sp_. Specifically, Figure 3(c) shows an *x*–*z* field map of |*E*_*z*_| at the left peak of Figure 3(b). We can directly see how the *E*_*z*_ field is distributed toward the PEC plate by $\left|f_{k_{z}}\right\rangle$ . As we have discussed in Figure 2, |*R*| ≈ 1 when *p*_*x*_ >> *k*_0_, meaning that $\left|f_{k_{z}}\right\rangle$ mostly resides in the form of evanescent waves having *k*_*z*_ >> *k*_0_. Within the first BA that suppresses coupling between $\left|f_{k_{z}}\right\rangle$ and $\left|a_{k_{z1}}\right\rangle$ when *k*_*z*_ ≠ *k*_*z*1_, this implies that the excitation of $\left|a_{k_{z}}\right\rangle$ inherits the same *k*_*z*_ distribution, and consequently the spatial decay of the field away from the two junctions follows the envelope defined by $\left|f_{k_{z}}\right\rangle$ . Because the separation between the two junctions is much smaller than the apparent spatial extent associated with $\left|f_{k_{z}}\right\rangle$ . Therefore, the proximity of the junctions relative to the evanescent‐decay distance ensures strong coupling mediated by $\left|a_{k_{z}}\right\rangle$ . For the right peak, the corresponding map is shown in Figure 3(d). Here, the two junctions are spaced by about one *λ*_sp_, so that the interaction mediated by $\left|a_{k_{z}}\right\rangle$ becomes negligibly weak. Consequently, the resonance appears at *a* = *a*_3_ with excellent agreement.

We note that there is no resonant behavior near *a* = *a*_2_, which is suppressed by the symmetry of the system and the normally incident planewave: breaking the symmetry allows a peak to emerge near *a* = *a*_2_ (see Supplementary Material).

## 5 Mapping to the metasurface-based heterostructures

So far, we have discussed the interaction between 2DSPs and the 1D junction formed by in-plane 2D crystal and a 2D PEC plane, with particular emphasis on an examination of the reflection coefficient *R* and consequent forming of a polariton-based resonant cavity. The heterostructure of 2D crystal and a PEC plane, however, is an idealized configuration that is not readily realizable in practice. Nevertheless, we note that it provides valuable intuition. Because the system is intrinsically two-dimensional, it can be directly mapped onto a metasurface platform, thereby yielding a practically feasible architecture. As discussed in Figure 3, inserting 2D crystal capable of supporting 2DSPs between two PEC planes suffices to realize an ultranarrow resonant cavity. The significance of this is that any structure that supports a bound state corresponding to the 2DSP within the gap between the PEC planes can serve as a resonant cavity. One straightforward way is to employ a metasurface that supports spoof surface polaritons (SSPs) via negative effective permittivity [30], [31], [32]. Consequently, Figure 3(a) conveys a clear and simple message: patterning the PEC plane with an appropriate SSP-supporting metastructure directly implements the desired resonant polaritonic cavity.

**Figure 4:**
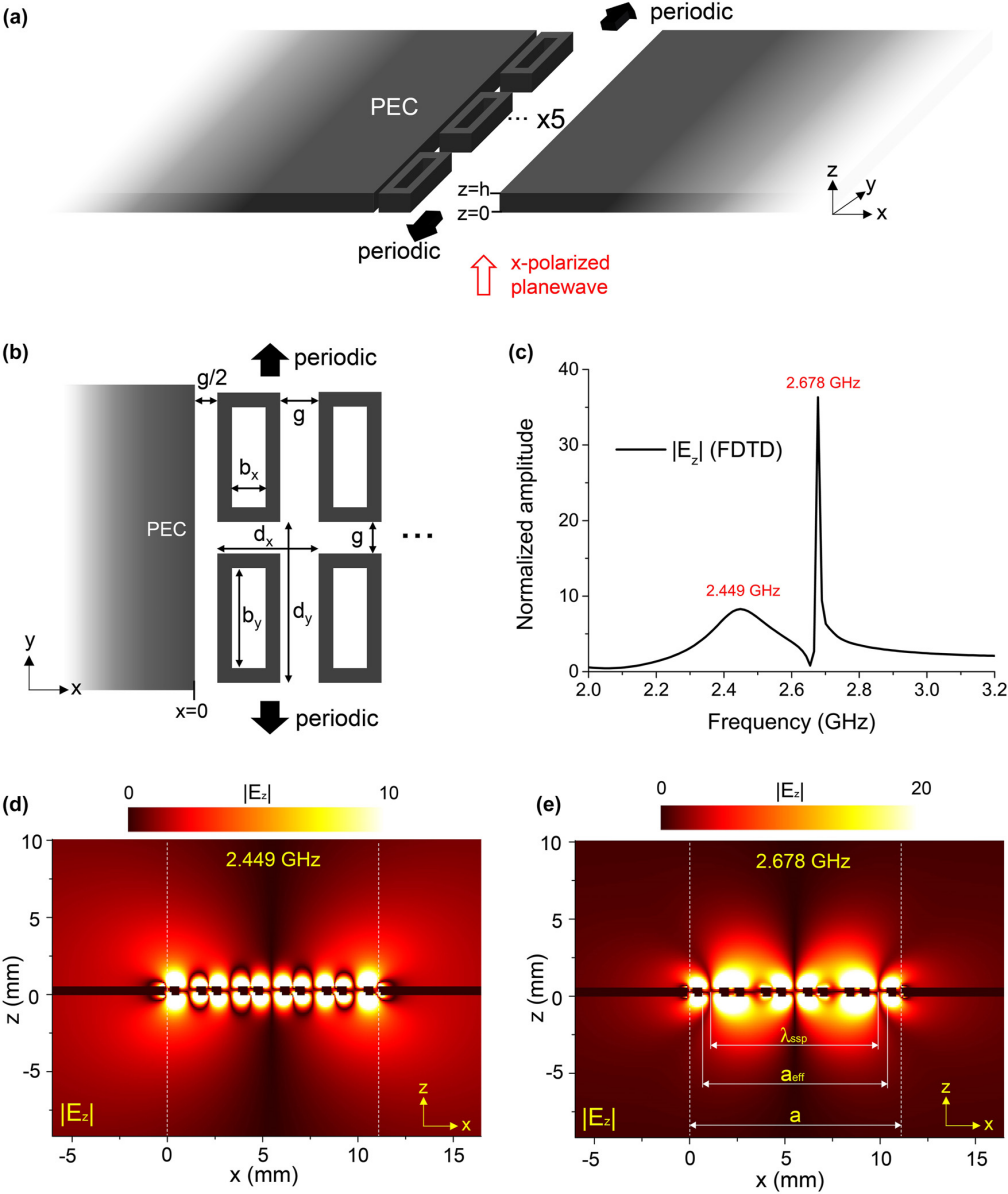
Metasurface-implementation of the resonant polaritonic cavity. (a) Schematic of the metasurface heterosystem. The system is periodic in the *y*-direction. Between the two PEC plates, five ring resonators are placed in the *x*-direction. The resonators are also made of PEC. The thickness *h* is set to be *h* = 0.5 mm. An *x*-polarized planewave is normally incident upon the system. (b) Detailed structural parameters of the embedded metasurface. We set *g* = 0.5 mm, *b*_*x*_ = 0.8 mm, *b*_*y*_ = 50 mm. The width of the resonators rim is 0.4 mm. (c) FDTD-calculated spectrum of |*E*_*z*_|, measured at just above the junction (*x* = 0, *z* = *h* ^+^). The amplitude is normalized by that of the incident. (d, e) FDTD-calculated *x*–*z* field maps. The *x*–*z* plane crosses the center of the ring resonator. (d) |*E*_*z*_| field maps at 2.449 GHz, corresponding to the first (*a*_1_) mode, (e) and at 2.678 GHz, corresponding to the third (*a*_3_) mode. Two vertical white dashed lines are the positions of the junctions. In (e), *λ*_ssp_ = 8.85 mm denotes the wavelength of SSPs, obtained by measuring the distance between two nodes near the junctions. *a*_eff_ = 9.70 mm is the effective cavity size, the distance between the inner left face of the leftmost ring resonator and the inner right face of the rightmost ring resonator.

Here we adopt a metasurface consisting of rectangular ring resonators, as shown in Figure 4(a) and (b). This metasurface is not only structurally simple but also suitable for placement between two PEC planes. The metasurface has been reported in prior studies to support negative effective permittivity and SSPs [33]. We find that using this metasurface heterostructure, a truncated metasurface combined with two PEC planes, revisits an earlier study that discussed the so-called fractional resonance [34]. In this work, we focus on implementing the polaritonic cavity using metasurfaces to verify our theory from a practical perspective.

The metasurface is truncated along the *x*-direction to contain only five resonators; it remains infinitely periodic along the *y*-direction. Adding more resonators in the *x*-direction could be considered, but this would increase the separation between the two PEC plates, which defeats the purpose of achieving an ultra-narrow cavity. Because each resonator behaves as an individual pixel, reducing the number of resonators would degrade the effective spatial resolution along the *x*-direction and consequently limiting the excitation of higher-order modes that require rapid spatial variation. By using five resonators, at least two distinct cavity modes can be supported as demonstrated in Figure 4(c). For the excitation of SSPs, we again used an *x*-polarized planewave incident normally upon the system from *z* < 0. In contrast to the Figure 3 case, where the spacing between the PEC planes was varying, we fixed the separation here. Instead, by using the intrinsic dispersive nature of the metasurface, we obtained the spectrum of |*E*_*z*_| using FDTD. |*E*_*z*_| is measured at *x* = 0 and *z* = *h* ^+^, and the result is shown in Figure 4(c). Two distinct peaks, a broader one at 2.449 GHz and a sharper one at 2.678 GHz, are clearly shown.

To verify whether the two peaks in Figure 4(c) correspond to the resonant cavity modes discussed in Figure 3, the most straightforward approach is to examine the field maps and determine the modal index of each peak. Using FDTD calculations, we obtained the *x*–*z* field distributions at the two relevant frequencies; these are shown in Figure 4(d) and (e). Figure 4(d) displays the field map for the broad peak at 2.449 GHz. In the near-field region of the metasurface, the strong influence of its geometry produces a highly intricate field pattern. However, as one moves away from the surface (increasing |*z*|), the distribution becomes similar to that of the *a*_1_ resonant mode observed in Figure 3(c), including the unique diffracting patterns in the PEC region. Figure 4(e) shows the field map corresponding to the sharp peak at 2.678 GHz, which similarly matches the *a*_3_ mode illustrated in Figure 3(d). Thus, we conclude that the broad and sharp peaks seen in Figure 4(c) correspond respectively to the *a*_1_ and *a*_3_ modes, confirming that a resonant polaritonic cavity has been realized through the metasurface-based heterostructure. Again, we note that the peak corresponding to *a*_2_ can be excited by breaking the symmetry (see Supplementary Material).

Our metasurface-based heterostructure has successfully demonstrated the feasibility of a resonant polaritonic cavity, with an extended frequency range. However, our analysis on the metasurface-based system is carried out phenomenologically using field maps, and a quantitative description of the correspondence to the 2D crystal/PEC system is still insufficient. Consequently, we examined the reflection phase shift of the SSPs in the metasurface-based system. Here, it should be noted that the embedded metasurface is truncated. Due to this finite-size effect, the effective permittivity retrieved from the infinitely wide metasurface cannot be employed to estimate SSP wavelength *λ*_ssp_. Instead, we directly extracted *λ*_ssp_ from the field map of the third peak (*a*_3_), as shown in Figure 4(e): we have *λ*_ssp_ = 8.85 mm. By using Eq. (8) with *a* = 11.0 mm and *m* = 3, we obtained arg(*R*) = 0.514*π*, which deviates from the theoretical value of 0.756*π* predicted by SLA. However, as shown in Figure 3(c) and (d), the junctions generate strong local fields whose maximum coincides precisely with the junction position, due to the charge accumulation through the lightning-rod effect. In contrast, for the metasurface case in Figure 4(e), the field maximum is shifted into the interior of the gap. Taking this observation into account, if we shift the junction location and define an *effective* cavity size *a*_eff_, the phase becomes 0.808*π*, which is remarkably close to the theoretical value. This demonstrates the quantitative feasibility of the metasurface-based system; however, because *a*_eff_ is not rigorously defined, further discussion on the physical meaning of the shifted junction is required.

## 6 Conclusions

In summary, we have theoretically investigated in-plane heterostructures composed of 2D crystals adjoining perfect electric conductor (PEC) planes, revealing their unique capability to support ultranarrow polaritonic resonant cavities. Our analysis demonstrates that at the one-dimensional junction between a 2D crystal and PEC plane, strongly confined two-dimensional surface polaritons (2DSPs) undergo near-total reflection with an anomalous phase shift of 3*π*/4. This represents a fundamental behavior of 2DSP reflection, characterized by a *π*/2 deviation from the conventional edge-reflection phase shift, signifying a distinct boundary condition intrinsic to the 2D system. The resulting anomalous reflection phase enables cavity resonance at dimensions as small as *λ*_sp_/8, substantially below the conventional *λ*_sp_/2 limit. Furthermore, by leveraging the spatial dimensionality of our heterostructure concept, we demonstrated a direct correspondence to practical metasurface implementations supporting spoof surface polaritons (SSPs), confirming that our theoretical framework extends beyond idealized 2D systems into practical structures operating in microwave and terahertz regimes. This straightforwardly implies that patterning a metallic plane with a metasurface possessing an effective negative permittivity readily realizes such ultranarrow resonators, whose operation can be intuitively understood in terms of SSPs.

Our work provides not only deeper insight into low-dimensional polariton optics but also establishes a new approach for designing phase-engineered polaritonic interfaces. The resulting ultranarrow resonant cavities hold substantial promise for applications including on-chip terahertz oscillators, electrically tunable infrared filters with enhanced spectral selectivity, and high-sensitivity molecular sensors operating at deeply subwavelength scales.

## Supplementary Material

Supplementary Material Details
