# Supplementary material for: Ultranarrow polaritonic cavities formed by one-dimensional junctions of two-dimensional in-plane heterostructures
Source: Nanophotonics. 2025 Nov 27;14(27):5417–25. doi: 10.1515/nanoph-2025-0467 (PMC12717917; doi:10.1515/nanoph-2025-0467)
Supplement: Supplementary file 1 — Supplementary Material Details [file j_nanoph-2025-0467_suppl_001.pdf]

Supplementary Material for

"Ultranarrow polaritonic cavities formed by one-dimensional junctions of two-dimensional in-plane heterostructures"

Seojoo Lee[1,2] and Ji-Hun Kang[3,4,5*]

[1] School of Applied and Engineering Physics, Cornell University, Ithaca, New York 13853, USA

[2] The Institute of Basic Science, Korea University, Seoul 02841, Republic of Korea

[3] Department of Optical Engineering, Kongju National University, Cheonan 31080, Republic of Korea

[4] Department of Future Convergence Engineering, Kongju National University, Cheonan 31080, Republic of Korea

[5] Institute of Application and Fusion for Light, Kongju National University, Cheonan 31080, Republic of Korea

Corresponding author: *jihunkang@kongju.ac.kr

1. The Coupled Integral Equations for *R* – Born Approximation

We start with Eq. (6) in the main text:

$$\begin{aligned} &(1-R)|p\rangle - \int_{-\infty}^{\infty} dk_z \alpha_{k_z} \left|a_{k_z}\right\rangle = \int_{-\infty}^{\infty} dk_z \phi_{k_z} \left| f_{k_z}\right\rangle \\ &p_x(1+R)|p\rangle + \int_{-\infty}^{\infty} dk_z k_x \alpha_{k_z} \left|a_{k_z}\right\rangle = \int_{-\infty}^{\infty} dk_z k_x \phi_{k_z} \left| f_{k_z}\right\rangle. \end{aligned} \tag{S1}$$

By projecting the first line of Eq. (S1) onto $\left| f_{k_z}\right\rangle$, we have

$$\phi_{k_z} = (1-R)W_{f,p}(k_z) - \alpha_{k_z} W_{f,a}(k_z) - \int_{-\infty}^{\infty} dk_{z1} \alpha_{k_{z1}} K_{f,a}(k_{z1};k_z), \tag{S2}$$

while projections of the second line of Eq. (S1) onto $\left|a_{k_z}\right\rangle$ and $|p\rangle$ respectively yield

$$\begin{aligned} &\alpha_{k_z} = \phi_{k_z} W_{a,f}(k_z) + \int_{-\infty}^{\infty} dk_{z1} \frac{k_{x1}}{k_x} \phi_{k_{z1}} K_{a,f}(k_{z1};k_z), \\ &1+R = \frac{1}{p_x}\int_{-\infty}^{\infty} dk_z k_x \phi_{k_z} W_{p,f}(k_z). \end{aligned} \tag{S3}$$

Here, $W_{p,q}(k_z) = W_{q,p}^{*}(k_z)$ and $K_{f,a}(k_{z1};k_z) = K_{a,f}^{*}(k_z;k_{z1})$. What we obtained in Eq. (S2) and (S3) are three coupled integral equations for *R*, *α*, and *ϕ*, governing the interaction between 2DSPs and the junction. Here, to deal with the integral equations, we adopt the Born approximation (BA) as follows.

For the zero-th approximation, let us start with the case of $\left|a_{k_z}\right\rangle = 0$, equivalent to the single-mode approximation in the crystal region. Then, Eq. (S2) and (S3) become

$$\phi_{k_z}^{(0)} = (1-R)W_{f,p}(k_z), \quad 1+R = \frac{1}{p_x}\int_{-\infty}^{\infty} dk_z k_x \phi_{k_z}^{(0)} W_{p,f}(k_z), \tag{S4}$$

where the superscript $^{(0)}$ denotes the zero-th approximation. From Eq. (S4), $R^{(0)}$ can be directly obtained as

$$R^{(0)} = \frac{I^{(0)}-1}{I^{(0)}+1},\ \ I^{(0)} \equiv \int_{-\infty}^{\infty} dk_z \frac{k_x}{p_x} \left| W_{p,f}\left(k_z\right) \right|^2. \tag{S5}$$

Now, we consider the first BA. By substituting the first line of Eq. (S3) into Eq. (S2), we have

$$\begin{aligned}
\phi_{k_z} &= \left(1-R\right) \frac{W_{f,p}\left(k_z\right)}{1+\left|W_{a,f}\left(k_z\right)\right|^2} \\
&- \frac{1}{1+\left|W_{a,f}\left(k_z\right)\right|^2} \int_{-\infty}^{\infty} dk_{z1} \phi_{k_{z1}} \left(\frac{k_{x1}}{k_x} W_{f,a}\left(k_z\right) K_{a,f}\left(k_{z1};k_z\right) + W_{a,f}\left(k_{z1}\right) K_{f,a}\left(k_{z1};k_z\right) \right) \\
&- \frac{1}{1+\left|W_{a,f}\left(k_z\right)\right|^2} \int_{-\infty}^{\infty} dk_{z2} \int_{-\infty}^{\infty} dk_{z1} \frac{k_{x1}}{k_{x2}} \phi_{k_{z1}} K_{a,f}\left(k_{z1};k_{z2}\right) K_{f,a}\left(k_{z2};k_z\right).
\end{aligned} \tag{S6}$$

The first BA is to account for only the first term of the right-hand-side of Eq. (S6) such that,

$$\phi_{k_z}^{(1)} = \left(1-R\right) \frac{W_{f,p}\left(k_z\right)}{1+\left|W_{a,f}\left(k_z\right)\right|^2}. \tag{S7}$$

This is equivalent to the suppression of dependency between $\left|f_{k_z}\right\rangle$ and $\left|a_{k_{z1}}\right\rangle$ for $k_{z1} \neq k_z$ (i.e., $K_{f,a}\left(k_{z1};k_z\right)=0$). By substituting Eq. (S7) into ϕ_{k_z} in the second line of Eq. (S3), we have

$$R^{(1)} = \frac{I^{(1)}-1}{I^{(1)}+1},\ \ I^{(1)} \equiv \int_{-\infty}^{\infty} dk_z \frac{k_x}{p_x} \frac{\left|W_{p,f}\left(k_z\right)\right|^2}{1+\left|W_{a,f}\left(k_z\right)\right|^2}. \tag{S8}$$

We can see that the first BA gives the correction term $\left|W_{a,f}\left(k_z\right)\right|^2$ to the denominator of the integrand of $I^{(0)}$ in Eq. (S5), exhibiting how the coupling between $\left|f_{k_z}\right\rangle$ and $\left|a_{k_{z1}}\right\rangle$ impacts the reflection. One can show that $I^{(1)}$ can be evaluated explicitly as

$$I^{(1)} = \left[\frac{4i}{\pi} \tanh^{-1}\left(\frac{-ip_z}{p_x} \right) - 2 \right] - \frac{\sqrt{k_0^2+p_x^2}}{p_x} \left[\frac{4i}{\pi} \tanh^{-1}\left(\frac{-ip_z}{\sqrt{k_0^2+p_x^2}} \right) - 2 \right]. \tag{S9}$$

2. Second Born Approximation

For the second Born approximation (BA), $\phi^{(2)}_{k_{z1}}$ can be obtained by replacing $\phi_{k_{z1}}$ in the integral parts of Eq. (S6) with $\phi^{(1)}_{k_{z1}}$. This gives rise to

$$\begin{aligned}\phi^{(2)}_{k_z} = \phi^{(1)}_{k_z} &- \frac{1}{1+\left|W_{a,f}\left(k_z\right)\right|^2}\int_{-\infty}^{\infty} dk_{z1} k_{x1} \phi^{(1)}_{k_{z1}} \int_{-\infty}^{\infty} dk_{z2} \frac{1}{k_{x2}} K_{a,f}\left(k_{z1};k_{z2}\right) K_{f,a}\left(k_{z2};k_z\right) \\ &- \frac{1}{1+\left|W_{a,f}\left(k_z\right)\right|^2}\int_{-\infty}^{\infty} dk_{z1} \phi^{(1)}_{k_{z1}} \left(\frac{k_{x1}}{k_x} W_{f,a}\left(k_z\right) K_{a,f}\left(k_{z1};k_z\right) + W_{a,f}\left(k_{z1}\right) K_{f,a}\left(k_{z1};k_z\right)\right).\end{aligned} \quad \text{(S10)}$$

Due to the complexity of the integral, our main objective here is to reduce the expression to a simpler form suitable for numerical evaluation. Let us first evaluate the k_{z2} integral part,

$$\begin{aligned}U\left(k_z,k_{z1}\right) &\equiv \int_{-\infty}^{\infty} dk_{z2} \frac{1}{k_{x2}} K_{a,f}\left(k_{z1};k_{z2}\right) K_{f,a}\left(k_{z2};k_z\right) \\ &= \frac{1}{4\pi^2}\int_{-\infty}^{\infty} dk_{z2} \frac{1}{k_{x2}} \frac{p_z^2}{p_z^2-k_{z2}^2} \frac{2ik_{z2}}{k_{z2}^2-k_z^2} \frac{2ik_{z2}}{k_{z1}^2-k_{z2}^2}.\end{aligned} \quad \text{(S11)}$$

The integral can be evaluated by using the contour integration in the complex plane with an appropriate choice of branch cuts. An alternative way is to use the Fourier transformation of Hankel function of the first kind. Specifically, we write

$$\frac{1}{k_{x2}} \equiv \frac{1}{\sqrt{k_0^2-k_{z2}^2}} = \frac{1}{2}\int_{-\infty}^{\infty} H_0^{(1)}\left(k_0\sqrt{z^2}\right) e^{-ik_{z2}z} dz. \quad \text{(S12)}$$

Then, Eq. (S11) can be rewritten as

$$U\left(k_z,k_{z1}\right) = \frac{1}{8\pi^2}\int_{-\infty}^{\infty} dz H_0^{(1)}\left(k_0\sqrt{z^2}\right)\int_{-\infty}^{\infty} dk_{z2} e^{-ik_{z2}z} \frac{p_z^2}{p_z^2-k_{z2}^2} \frac{2ik_{z2}}{k_{z2}^2-k_z^2} \frac{2ik_{z2}}{k_{z1}^2-k_{z2}^2}. \quad \text{(S13)}$$

Note that the integral must be evaluated in the sense of Cauchy's principal values. Accordingly, the k_{z2} integration in Eq. (S13) can be carried out as follows:

$$U\left(k_z,k_{z1}\right)=-\frac{p_z^2}{2}\int_{-\infty}^{\infty}dzH_0^{(1)}\left(k_0\sqrt{z^2}\right)$$
$$\times\left[\frac{e^{ip_z|z|}ip_z}{\left(k_{z1}^2-p_z^2\right)\left(k_z^2-p_z^2\right)}+\frac{\frac{z}{|z|}\sin\left(k_z z\right)k_z}{\left(k_z^2-p_z^2\right)\left(k_{z1}^2-k_z^2\right)}-\frac{\frac{z}{|z|}\sin\left(k_{z1} z\right)k_{z1}}{\left(k_{z1}^2-p_z^2\right)\left(k_{z1}^2-k_z^2\right)}\right]. \quad \text{(S14)}$$

The integral is now changed to three spatial integrals involving Hankel function of the first kind, exponential, and sine functions. Direct evaluation of Eq. (S14) yields

$$U\left(k_z,k_{z1}\right)$$
$$=-\frac{2ip_z^2}{\pi}\left[\frac{\frac{p_z}{\sqrt{k_0^2-p_z^2}}\arccos\left(\frac{p_z}{k_0}\right)}{\left(k_{z1}^2-p_z^2\right)\left(k_z^2-p_z^2\right)}+\frac{\frac{k_z}{\sqrt{k_0^2-k_z^2}}\arcsin\left(\frac{k_z}{k_0}\right)}{\left(k_z^2-p_z^2\right)\left(k_{z1}^2-k_z^2\right)}-\frac{\frac{k_{z1}}{\sqrt{k_0^2-k_{z1}^2}}\arcsin\left(\frac{k_{z1}}{k_0}\right)}{\left(k_{z1}^2-p_z^2\right)\left(k_{z1}^2-k_z^2\right)}\right]. \quad \text{(S15)}$$

Now, we rewrite Eq. (S10) as

$$\phi_{k_z}^{(2)}=\phi_{k_z}^{(1)}-\frac{1}{1+\left|W_{a,f}\left(k_z\right)\right|^2}\int_{-\infty}^{\infty}dk_{z1}k_{x1}\phi_{k_{z1}}^{(1)}U\left(k_z,k_{z1}\right)$$
$$-\frac{1}{1+\left|W_{a,f}\left(k_z\right)\right|^2}\int_{-\infty}^{\infty}dk_{z1}\phi_{k_{z1}}^{(1)}\left(\frac{k_{x1}}{k_x}W_{f,a}\left(k_z\right)K_{a,f}\left(k_{z1};k_z\right)+W_{a,f}\left(k_{z1}\right)K_{f,a}\left(k_{z1};k_z\right)\right). \quad \text{(S16)}$$

Putting Eq. (S16) into the right-hand-side of the second line of Eq. (S3) gives rise to

$$\frac{1}{p_x}\int_{-\infty}^{\infty}dk_zk_x\phi_{k_z}^{(2)}W_{p,f}\left(k_z\right)=\left(1-R\right)I^{(1)}-\frac{1}{p_x}\int_{-\infty}^{\infty}dk_zk_x\frac{W_{p,f}\left(k_z\right)}{1+\left|W_{a,f}\left(k_z\right)\right|^2}\int_{-\infty}^{\infty}dk_{z1}k_{x1}\phi_{k_{z1}}^{(1)}U\left(k_z,k_{z1}\right)$$
$$-\frac{1}{p_x}\int_{-\infty}^{\infty}dk_zk_x\frac{W_{p,f}\left(k_z\right)}{1+\left|W_{a,f}\left(k_z\right)\right|^2}\int_{-\infty}^{\infty}dk_{z1}\phi_{k_{z1}}^{(1)}\left(\frac{k_{x1}}{k_x}W_{f,a}\left(k_z\right)K_{a,f}\left(k_{z1};k_z\right)+W_{a,f}\left(k_{z1}\right)K_{f,a}\left(k_{z1};k_z\right)\right).$$

(S17)

We note that the two integral parts in the last term of Eq. (S17) are the same. Specifically, one can readily find that

$$\frac{1}{p_x}\int_{-\infty}^{\infty}dk_z k_x \frac{W_{p,f}(k_z)}{1+\left|W_{a,f}(k_z)\right|^2}\int_{-\infty}^{\infty}dk_{z1}\phi_{k_{z1}}^{(1)}\frac{k_{x1}}{k_x}W_{f,a}(k_z)K_{a,f}(k_{z1};k_z)$$
$$=\frac{1}{p_x}\int_{-\infty}^{\infty}dk_z k_x \frac{W_{p,f}(k_z)}{1+\left|W_{a,f}(k_z)\right|^2}\int_{-\infty}^{\infty}dk_{z1}\phi_{k_{z1}}^{(1)}W_{a,f}(k_{z1})K_{f,a}(k_{z1};k_z) \tag{S18}$$
$$=-\frac{2(1-R)}{\pi}\left(\frac{p_z}{\sqrt{2}p_x}-\left[2i\tanh^{-1}\left(\frac{-ip_z}{p_x}\right)-\pi\right]+\left[\frac{\alpha}{p_x}-\frac{k_0^2}{\sqrt{2}\alpha p_x}\right]\left[2i\tanh^{-1}\left(\frac{-ip_z}{\alpha}\right)-\pi\right]\right),$$

with $\alpha\equiv\sqrt{2k_0^2-p_z^2}=\sqrt{k_0^2+p_x^2}$. Finally, we arrive at the reflection coefficient $R^{(2)}$ with the second BA, which is given by

$$R^{(2)}=\frac{I^{(2)}-1}{I^{(2)}+1},$$
$$I^{(2)}\equiv I^{(1)}-\frac{1}{p_x}\int_{-\infty}^{\infty}dk_z k_x \frac{W_{p,f}(k_z)}{1+\left|W_{a,f}(k_z)\right|^2}\int_{-\infty}^{\infty}dk_{z1}k_{x1}\frac{W_{f,p}(k_{z1})}{1+\left|W_{a,f}(k_{z1})\right|^2}U(k_z,k_{z1}) \tag{S19}$$
$$+\frac{4}{\pi}\left(\frac{p_z}{\sqrt{2}p_x}-\left[2i\tanh^{-1}\left(\frac{-ip_z}{p_x}\right)-\pi\right]+\left[\frac{\alpha}{p_x}-\frac{k_0^2}{\sqrt{2}\alpha p_x}\right]\left[2i\tanh^{-1}\left(\frac{-ip_z}{\alpha}\right)-\pi\right]\right).$$

The remaining integral term in the coupling factor $I^{(2)}$ can be evaluated numerically.

3. Excitation of the second resonance modes

The excitation of resonance modes supported by a cavity can be suppressed by symmetry. In our system, as discussed in Figs. 3 and 4, the second resonance mode (m = 2) was not excited due to the symmetry of both the system and normally incident light. The simplest way to excite this mode is to break the symmetry. If the system itself is fixed, this can be achieved by breaking the symmetry of the incident light. In other words, instead of using normally incident light, a different form of incidence can be employed. Here, we use a point source located at the lower-left side of the system to generate a circular electric wave. In the case of a metasurface-based system, a line source extended along the y-axis is used to generate a cylindrical electric wave. As shown

in Fig. S1, the second resonance modes, which were suppressed in the original systems with preserved symmetry, are successfully excited here.

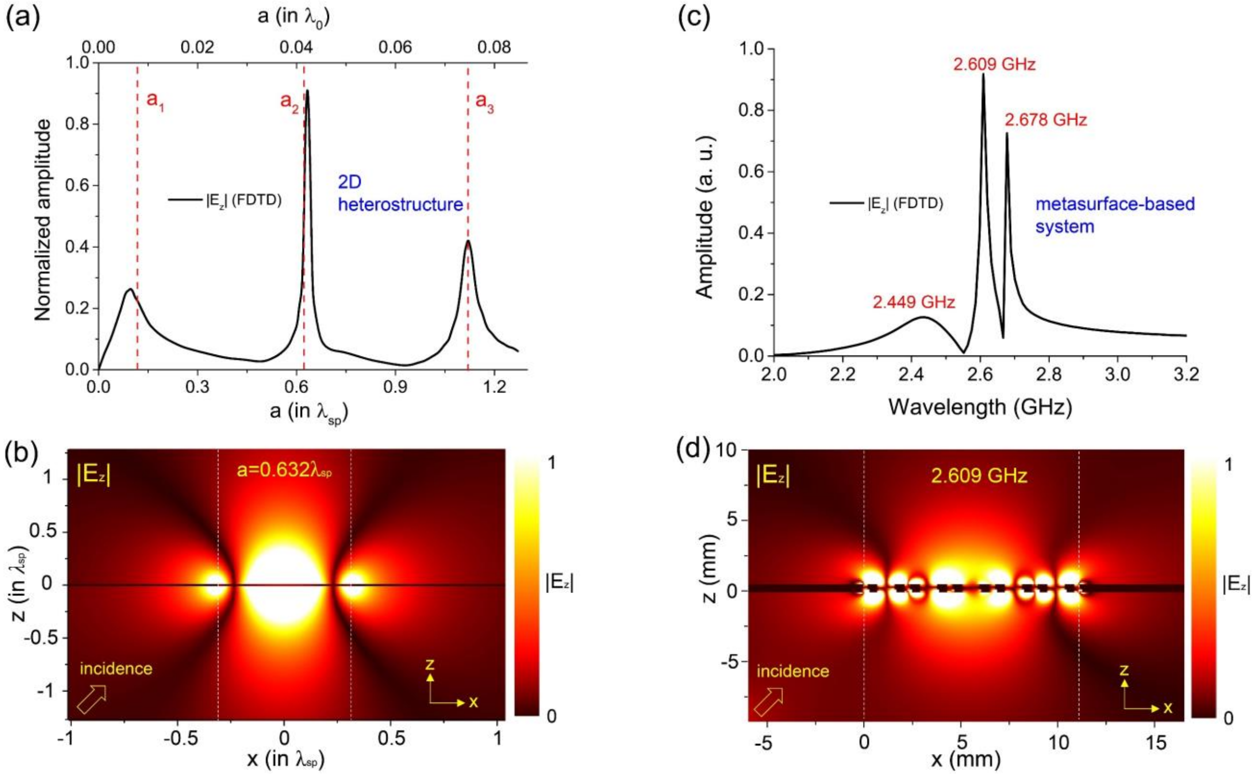

Fig. S1. Excitation of the second modes by breaking the symmetry of the incident light. (a) FDTD-calculated $|E_z|$ of the 2D heterostructure with varying a, and (b) the corresponding x-z field map of the second mode (a_2). The incident light was generated by a point magnetic source located at $(x, z) = (-2\lambda_{sp}, -2\lambda_{sp})$. (c) FDTD-calculated spectrum of $|E_z|$ of the metasurface-based system, and (d) the corresponding field map of the second mode at 2.609 GHz. For (c) and (d), the incident light was generated by a line magnetic source, which is infinitely extended along the y-direction and located at $(x, z) = (-10$ mm, -15 mm). All structures are identical to those used in Fig. 3 and 4 of the main text.

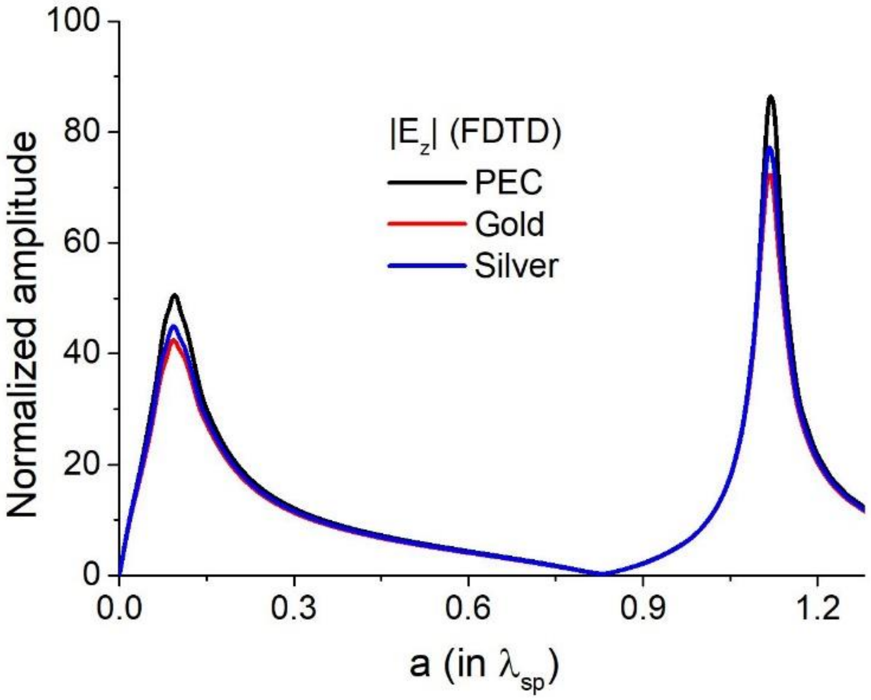

Fig. S2. FDTD-calculated resonant polaritonic cavity. The PEC plane of the original system is replaced by a real metal plane. We set λ_0 = 6 μm, λ_{sp} = 400 nm, and h = 4 nm where h is the thickness of the 2D crystal and metal planes.
